# Weaker Functional Connectivity Strength in Patients with Type 2 Diabetes Mellitus

**DOI:** 10.3389/fnins.2017.00390

**Published:** 2017-07-07

**Authors:** Linlin Liu, Wanhu Li, Yang Zhang, Wen Qin, Shan Lu, Quan Zhang

**Affiliations:** ^1^Department of Radiology and Tianjin Key Laboratory of Functional Imaging, Tianjin Medical University General HospitalTianjin, China; ^2^Department of Radiology, Shandong Cancer Hospital Affiliated to Shandong University, Shandong Academy of Medical SciencesJinan, China; ^3^Department of Radiology, Tianjin Medical University Metabolic Diseases HospitalTianjin, China

**Keywords:** type 2 diabetes mellitus, resting state functional connectivity strength, insula, supramarginalgyrus, fMRI

## Abstract

Type 2 diabetes mellitus (T2DM) is related to cognitive impairments and increased risk for dementia. Neuroimaging studies have demonstrated T2DM-related brain structural and functional changes which are partly associated to the cognitive decline. However, few studies focused on the early neuroimaging findingsin T2DM patients. In this study, a data-driven whole-brain resting state functional connectivity strength (rsFCS) methodwas used to evaluate resting functional changes in 53 T2DM patients compared with 55 matched healthy controls (HCs), and to detect the associations between the rsFCSchanges and cognitive functions in T2DM patients. The T2DM patients exhibited weaker long-range rsFCS in the right insula and weaker short-range rsFCS in the right supramarginalgyrus (SG) compared with the HCs. Additionally, seed-based functional connectivity (FC) analysis revealed weaker FC between the right insula and the bilateral superior parietal lobule (SPL), and between the right SG and the bilateral supplementary motor area (SMA)/right SPL in T2DM patientscompared with the HCs. In T2DM patients, negative correlation was found between the long-range rsFCS in the right insula and HbA1c levels; and the FC between the right SG and the bilateral SMA negatively correlated with TMT-A scores. Our results indicated that the rsFCS alteration occurredbefore obvious cognitive deficits in T2DM patients, which might be helpful for understanding the neuromechanism of cognitive declines in T2DM patients.

## Introduction

Diabetes mellitus is one of the most common chronic metabolic diseases. Type 2 diabetes mellitus (T2DM) is associated with cognitive decline (Gispen and Biessels, 2000; Cheng et al., 2012), and increases risk for dementia, including vascular dementia, and Alzheimer's diseases (Janson et al., 2004; Tan et al., 2011; Haight et al., 2015). Increasing neuroimaging studies have focused on the neuromechanisms underling the cognitive impairments in T2DM patients, and T2DM-related brain structural and functional abnormalities have been revealed. For example, cerebral atrophy (De Bresser et al., 2010) and gray matter volume (GMV) reductions (Kumar et al., 2008; Moran, 2013) have been reported in patients with T2DM. Additionally, T2DM patients have also exhibited altered brain activity during working memory tasks (Ran-Ran Huang et al., 2015; Wood et al., 2016) and resting state (Chen et al., 2015a; Cui et al., 2015).

The resting state fMRI (rsfMRI) was widely used to evaluate regional brain activity and functional connectivity under disease conditions (Greicius, 2008; Barkhof et al., 2014; Liu et al., 2015a). Recently, researchers have focused on the T2DM-related brain function alteration at resting state. For example, the altered spontaneous brain activity in multiple brain regions has been revealed by both low-frequency fluctuations (ALFF; Xia et al., 2013; Cui et al., 2014; Wang C. X. et al., 2014; Zhou et al., 2014) and regional homogeneity (ReHo) studies (Cui et al., 2014; Peng et al., 2016) on T2DM patients. The ALFF and ReHo analysis are two most important methods to describe the characteristics of rsfMRI signals in the brain. ALFF algorithm is to obtain the power spectrum by Fourier transform of the fMRI data, and then calculates the low-frequency oscillation amplitude of each voxel. It is measuring the strength of neural activity at voxel level (Zang et al., 2007). While ReHo is based on the calculation of the Kendall harmonious coefficient to measure the neural synchrony between a specific voxel and its neighboring voxel (Zang et al., 2004). The independent component analysis (ICA) method contributes to separate the whole brain signal from physiological noise and automatically captures the entire network as a single major component (Greicius et al., 2003; Liu et al., 2017). By using ICA, resting state FC within default mode network (DMN) and attention network (AN) were found to be disrupted in T2DM patients (Cui et al., 2015; Xia et al., 2015a; Yang et al., 2016); and the FC alterations were found to be related to visualspatial function (Cui et al., 2015; Xia et al., 2015a) and working memory ability (Xia et al., 2015a). Seed-based resting state FC method was also used for detecting brain FC changes in T2DM patients. It is very important for FC method to select the seed, which is usually based on a priori anatomical knowledge or activation graph (Lowe et al., 2002). With Seed-based resting state FC, inconsistent results were obtained in T2DM patients. For example, Chen et al. (2014) reported that T2DM patients exhibited decreased FC between the posterior cingulate cortex (PCC) and the right middle temporal gyrus (MTG), the left occipital cortex and the left precentral region; and increased FC between the PCC and the right frontal regions. Another study by Chen et al (Chen et al., 2015b) revealed decreased FC between the thalamus and the right MTG and the cuneus in T2DM patients; and these decreased FC were associated with executive function and visualspatial ability. A study by Musen et al. (2012) demonstrated decreased FC between the PCC and the frontal, temporal regions and the bilateral thalamus in T2DM patients. Additionally, altered FC between hippocampi and wide frontal, temporal and occipital regions were also reported in T2DM patients (Zhou et al., 2010; Xia et al., 2014).

These rsfMRI studies indicated that the alterationof FC was associated with the T2DM-related cognitive decline and deepened the understanding of neuromechanisms underling the cognitive impairments in T2DM patients. However, T2DM patients enrolled in most of these studies either companied by complications such as diabetic retinopathy, nephropathy, or peripheral neuropathy (Cui et al., 2014; Chen et al., 2015b) or suffered from cognitive impairments (Chen et al., 2014, 2015a,b; Cui et al., 2014; Wang C. X. et al., 2014; Xia et al., 2014) and neuroimaging findings based on these studies could not reflect the early alterations of brain function in T2DM patients. Only one rsfMRI study by Musen et al. (2012) focused on thecognitively intact T2DM patients and demonstrated that reduced FC in several regions within DMN were earlier than occurrence of cognition decline, which indicated the neuroimaging might be used for predicting the early cognitive impairments in T2DM patients.

Furthermore, previous studies demonstrated that functional brain changes in T2DM patients were widespread across the whole brain. However, ALFF and ReHo were methods for evaluating the regional resting brain activity (Zang et al., 2004, 2007); and the resting state FC is a hypothesis-driven seed-based method and cannot be used to evaluate the FC among the whole brain (Rosazza and Minati, 2011). The resting state functional connectivity strength (rsFCS) is a data-driven method for detecting functional connectivity among the whole brain andhas been proposed to reflect intrinsic functional organization of the brain (Zhuo et al., 2014), and can be used to further examine the association between the FC and anatomical distance (Guo et al., 2015). A systematical human brain activity relied on both long- and short -range FC (Sepulcre et al., 2010). Long-range FC is the way of high metabolic and time costs to perform (Bullmore and Sporns, 2012; Liang et al., 2013), while short-range FC is the way of low metabolic and time costs (Raymond et al., 2005). Therefore, the rsFCS method is suitable for evaluating the T2DM-related brain alterations in terms of whole brain FC.

In this study, a data-driven whole-brain rsFCS method was used to evaluate the resting functional brain changes in 53 T2DM patients compared with 55 matched healthy controls (HCs). Additionally, the clusters with significant intergroup difference in rsFCS were defined as the seeds, and seed-based FC analysis was further conducted to explore the specific brain regions among which the FC changes leading to the rsFCS alterations. Correlation analysis was performed between altered FCS/FC and clinical/cognitive variables. We aimed to explore the early rsFCS alterations in T2DM patients, which may be helpful for understanding the neuromechanism of cognitive declines in T2DM patients.

## Methods

### Subjects

Fifty-nine right-handed T2DM patients from endocrinology department of Tianjin Medical University General Hospital and 59 matched euglycemia healthy controls (HCs) were recruited in our study. All of the patients were diagnosed as T2DM according to the 2010 criteria of the ADA (2010), and none of them had any T2DM-related complications including diabetic retinopathy, diabetic nephropathy or peripheral neuropathy. The diagnostic criteria and classification standard of retinopathy, hypoglycemia and neuropathy were all based on China Diabetes Society Special Issue Standards of care for type 2 diabetes in China-2010 version. The presence of retinopathy was ascertained using direct ophthalmoscopy performed by ophthalmologist; the peripheral neuropathy was determined by clinical examination; the nephropathy by laboratory test of microalbuminuria. All of the patients have no clinical record of severe hypoglycemia during the last 2 years, and self-reported no experience of severe hypoglycemia (such as developing a symptom of dizziness or confusion). Twenty-seven of the 59 T2DM patients controlled blood glucose with oral hypoglycemic agents; ten patients were under treatment with insulin; thirteen patients were treated with both insulin and oral hypoglycemic agents; and nine patients were treated with only moderate exercise and diet therapy.

Exclusion criteria for both groups included: (1) Mini-Mental State examination (MMSE) score <27; (2) psychiatric or neurologic disorders that could influence cognitive functions; (3) cerebrovascular accidents (screened by history and MR scan); (4) self-reported history of alcohol or substance abuse; (5) family history of dementia.

The protocol of this study was approved by the Ethical Committee of Tianjin Medical University General Hospital, and all of the participants provided written informed consent according to institutional guidelines.

### Clinical data and neuropsychological tests

Clinical data were collected, including weight, height, and blood pressure. Body mass index (BMI) was calculated as weight in kilograms divided by the square of height in meters. Blood pressure was measured while sitting at three different time points during the day and averaged. Blood samples were obtained after an overnight fast of at least 10 h to test the levels of fasting blood glucose (FBG), triglycerides (TG), total cholesterol (TC), low density lipoprotein cholesterol (LDL), high density lipoprotein cholesterol (HDL) and glycated hemoglobin (HbA1c).

A battery of neuropsychological tests was used to evaluate participants' general mental status and cognitive domains. The Mini-Mental State Exam (MMSE) was used to assess possible dementia (Folstein et al., 1975). The anxiety and depression were evaluated with Self-Rating Anxiety Scale (Zung, 1971) and Self-Rating Depressive Scale, respectively (Zung, 1965). The Auditory Verbal Learning Test (Rosenberg et al., 1984) was used to test short-term and long-term memory. Working memory was assessed with the forward and backward digit span tests (Powell and Hiatt, 1996). Information process speed was tested with trail making test A (TMT-A; Reitan, 2011). A modified version (Zhang et al., 2015) of the Attention Network Test reported by Fan et al. (2002) was used to evaluate attentional function. Executive function was evaluated with the Wisconsin Card Sorting Test (Grant and Berg, 1948). Raven's Standard Progressive Matrices was used to test the intelligence quotient (IQ).

### MRI data acquisition

MRI data were acquired using a 3.0-Tesla MR scanner (Discovery MR750, GE Milwaukee, WI, USA). During scanning, foam pads and headphones were used to control head motion and decrease scanner noise as much as possible. The conventional T2 weighted images (T2WI), used to exclude the visible brain lesions, were acquired using a fast spin echo sequence with the following parameters: Repetition time (TR) = 3,400 ms, echo time (TE) = 85 ms, flip angle (FA) = 90°, field of view (FOV) = 256 mm × 256 mm, matrix = 256 × 256, slice thickness = 3 mm, slice gap = 1 mm. Sagittal three-dimensional T1-weighted images (3D-T1WI) were acquired by a brain volume (BRAVO) sequence with the following parameters: TR = 8.2 ms, TE = 3.2 ms, inversion time (TI) = 450 ms, FA = 12°, FOV = 256 mm × 256 mm, matrix = 256 × 256, slice thickness = 1 mm, no gap, and 188 sagittal slices. Resting-state functional BOLD images were acquired using a gradient-echo single shot echo planar imaging sequence with the following parameters: TR/TE = 2,000/45 ms, FOV = 220 mm × 220 mm, matrix = 64 × 64, FA = 90°, slice thickness = 4 mm, gap = 0.5 mm, 32 interleaved transverse slices, and 180 volumes. During fMRI scanning, all of participants were required to keep their eyes closed, relax, move as little as possible, think of nothing in particular, and not fall asleep during the fMRI scans. Before each fMRI acquisition, ten dummy scans were performed to allow the fMRI signals to reach a steady state.

### Data preprocessing and metrics calculations

Resting-state fMRI data were preprocessed using Statistical Parametric Mapping software (SPM8; http://www.fil.ion.ucl.ac.uk/spm). The first 10 volumes of each participant were deleted to allow the signal to reach equilibrium. The 170 remaining volumes were preprocessed with the following steps: correcting acquisition time delay between slices, head motion correction, regressing out nuisance covariates, temporal filtering (0.01–0.08 Hz), and normalization. The images were not smoothed so as to avoid introducing artificial local spatial correlation (Liu et al., 2015b). Nuisance covariates included six motion parameters, their first time derivations, and average BOLD signals of the cerebral spinal fluid and white matter. The global mean signal was not regressed out from the data (Hahamy et al., 2014). Next, the individual structural images were linearly registered to the mean functional images; the structural images were then linearly co-registered to Montreal Neurological Institute (MNI) space. Finally, each filtered functional volume was spatially normalized to MNI space using co-registration parameters and resampled into a voxel size of 3 × 3 × 3 mm^3^. Six T2DM patients and four HCs were excluded because of obvious head movement of larger than 2 mm translation in any axis or 2° rotation in any axis during fMRI scanning. Finally, the data from 53 T2DM patients and 55 HCs were included in analysis.

Whole-brain FC analysis were performed for the preprocessed data, and Pearson's correlation coefficients were calculated between the time series of each voxel and the other voxels within a whole brain gray matter mask (SPM8's gray matter probability template with a threshold of >0.3) (Feng et al., 2015), and a FC matrix was obtained for each participant. Because of the issue related to negative correlations on FC remains controversial (Murphy et al., 2009; Chai et al., 2012), we only performed analysis on long- and short-range positive rsFCS. Consistent with the previous study (Wang L. et al., 2014), a threshold of 0.2 was used to eliminate weak correlations possibly arising from signal noise. Then, the FC matrix was converted in to z-score using Fisher's z transformation to improve the normality. The rsFCS for a given voxel was calculated as the sum of the connectivities (*z*-values) between this voxel and all other voxels. Based on the anatomical distance, the rsFCS was divided into long- and short-range rsFCS (Guo et al., 2015). The long-range rsFCS of a given voxel was defined as the sum of the connectivities (*z*-values) between this voxel and other voxels with Euclidean distance of >75 mm from the MNI coordinate of this voxel, while the short-range rsFCS of a voxel was defined as the sum of the connectivities (*z*-values) with Euclidean distances of <75 mm (Achard et al., 2006; He et al., 2007). Finally, long- and short-range rsFCS maps were spatially smoothed using a Gaussian kernel of full-width at half-maximum (FWHM) of 6 mm. To determine whether the stability of rsFCS analysis was affected by the selection of correlation thresholds (0.2), rsFCS maps were also calculated based on two additional correlation thresholds (0.1 and 0.3).

To further examine the validity of the findings in the rsFCS analysis, a seed-based FC analysis was conducted. The seed was established as a sphere (6 mm in diameter) region of interest (ROI) centering at the peak coordinate of each cluster with a significant intergroup difference in long- or short-rang rsFCS. Pearson correlations were performed in each participant between the seed ROI and the other voxels within the gray matter masks. And then, a Fisher z-transform was applied to improve the normality of the correlation coefficients.

Previous study reported that GMV changes impact the FC (Bäuml et al., 2014; Kraus et al., 2014). Therefore, intergroup comparisons in GMV were also performed in this study. Three-dimension T1WI data were preprocessed using VBM8 tool-box (http://dbm.neuro.uni-jena.de/vbm/) of the SPM8 (Well-come Department of Imaging Neuroscience, London, UK; available at http://www.fil.ion.ucl.ac.uk/spm/software/spm8) implemented on MATLAB R2010a (Math Works Inc., Sherborn, MA, USA). First, brain tissues were segmented into gray matter, white matter, and cerebral spinal fluid and the gray matter segments were normalized to the MNI template by diffeomorphic anatomical registration through exponentiated lie algebra (DARTEL). The normalized GM segments were then modulated by dividing the Jacobian of the warp field to correct for local expansion or contraction (Liu et al., 2012). Finally, modulated gray matter images were smoothed with an isotropic Gaussian kernel of 8 mm full-width at half maximum.

### Statistical analysis

SPSS 21.0 (SPSS, Inc, Chiago.IL) was used to conduct the statistical analysis. Kolmogorov-Smirnovb test was performed to test normality of the demographic and clinical variables. For the normally distributed variables, the two-tailed independent samples *t*-tests were used. Non-normally distributed data were evaluated using the Mann-Whitney *U*-test. A chi-squared (χ^2^) test was used to assess intergroup difference in gender. The significant level was set as *P* < 0.05.

Voxel-wise two-sample *t*-test embedded in SPM8 was performed to exam the intergroup differences in the long- and short-range rsFCS after controlling for gender, age and years of education. Moreover, the same statistical steps were applied to rsFCS maps at the thresholds of 0.1 and 0.3.

The intergroup differences in the seed-based FC were tested using voxel-wise two-sample *t*-test embedded in SPM8 after controlling for age, gender and years of education (*P* < 0.05, AlphaSim correction). The clusters with intergroup difference in FC were also established as ROIs.

The ROI-based partial correlation analyses were conducted between rsFCS/FC and clinical/cognitive variables in the T2DM and HCs groups after controlling for age, gender and years of education. The significant level was set as *P* < 0.05. Bonferroni correction was used to control the multiple comparisons. Besides, the differences in correlation coefficients between the two groups were also assessed by *z*-test (*P* < 0.05, uncorrected).

The intergroup difference in GMV within whole brain was tested using voxel-based two-sample *t*-test embedded in SPM8 after controlling for age, gender and years of education.

Multiple comparisons correction for the intergroup compasions of rsFCS, seed-based FC and the GMV were performed using a Monte Carlo simulation (AlphaSim program in REST software, http://www.restfmri.net. Parameters: individual voxel *P* = 0.001, 1,000 simulations, smoothing kernel estimated using the T map, edge connected; with a global gray matter mask).

## Results

### Demographics and clinical results

Demographic, clinical and cognitive information of the T2DM patients and HCs are listed in Table 1. There were no significant intergroup differences in demographics or cognitive performance (*P* > 0.05). Compared with the controls, the patients had increased FBG (P < 0.001) and HbA1c (P < 0.001) levels.

**Table 1:** Demographics, clinical data, cognitive assessment.

	**Diabetic patients (*n* = 53)**	**Healthy controls (*n* = 55)**	**Statistics**	***P*-value**
**DEMOGRAPHICS**				
Age (years)	56.23 ± 7.72	55.16 ± 7.05	*t* = 0.748	0.456
Gender (M/F)	25/28	29/ 26	*χ^2^ =* 0.333	0.564
Education (years)	11.36 ± 3.24	10.87 ± 2.72	*t* = 0.846	0.400
**CLINICAL DATA**				
Disease duration (years)	8.02 ± 6.11	–	–	–
Weight (kg)	70.26 ± 8.80	68.35 ± 9.78	*t =* 1.065	0.289
Height (cm)	165.66 ± 7.79	166.07 ± 6.77	*t =* −0.294	0.769
BMI (kg/m^2^)	25.51 ± 2.39	24.71 ± 2.69	*t* = 1.635	0.105
Systolic BP (mmHg)	131.8 (110, 170)	127.5 (100, 170)	*z* = −1.885	0.059
Diastolic BP (mmHg)	83.1 (60, 115)	82.4 (60, 100)	*z* = −0.497	0.619
Total Cholesterol (mmol/L)	5.02 ± 1.06	5.33 ± 0.82	*t* = −1.639	0.104
Triglycerides (mmol/L)	1.90 ± 1.09	1.54 ± 1.05	*t* = 1.677	0.097
LDL (mmol/L)	3.17 ± 0.99	3.41 ± 0.79	*t* = −1.371	0.174
HDL (mmol/L)	1.17 ± 0.27	1.24 ± 0.25	*t* = −1.272	0.206
HbA1c (%)	8.60 ± 2.05	5.60 ± 0.35	*t =* 10.406	<0.001
HbA1c (mmol/mol)	70.49 ± 22.32	37.83 ± 3.83	*t =* 10.399	<0.001
FBG (mmol/L)	8.33 ± 2.70	5.13 ± 0.67	*t =* 8.282	<0.001
**COGNITIVE ASSESSMENT**				
MMSE score	29.12 ± 0.98	29.35 ± 0.98	*t =* −1.194	0.235
SAS	33.58 ± 6.94	32.17 ± 5.36	*t =* 1.154	0.251
SDS	34.87 ± 8.68	32.94 ± 7.32	*t =* 1.221	0.225
Raven score	40.73 ± 10.74	41.36 ± 9.46	*t =* −0.302	0.763
ACC (%)	98.55 ± 2.00	98.11 ± 4.40	*t =* 0.643	0.522
RT (ms)	540.33 ± 77.96	567.88 ± 95.04	*t =* −1.571	0.119
TMT-A (s)	62.68 ± 27.22	58.95 ± 28.46	*t =* 0.655	0.514
RPEP (%)	0.06 ± 0.03	0.06 ± 0.02	*t =* −0.231	0.798
Short-term memory	46.06 ± 9.68	47.64 ± 10.19	*t =* −0.795	0.429
Long-term memory	10.47 ± 2.90	10.44 ± 3.04	*t =* −0.049	0.961
Forward digit span	8.22 ± 1.45	8.28 ± 1.23	*t =* 0.223	0.824
Backward digit span	5.06 ± 1.53	5.14 ± 1.21	*t =* −0.290	0.773

*Values distributed normally or non-normally are presented as Mean ± SD or median (minimum, maximum). ACC, the accuracy rate of Attention Network Test; FBG, fasting blood glucose; HDL, high density lipoprotein; LDL, low density lipoprotein; MMSE, mini-mental state examination; RPEP (%), the percentage of the preservative response error of the Wisconsin Card Sorting Test; RT, the reaction time of Attention Network Test; SAS, self-rating anxiety scale; SDS, self-rating depression sale; TMT-A, trail making test-A*.

### Intergroup differences in rsFCS

**Figure 1:**
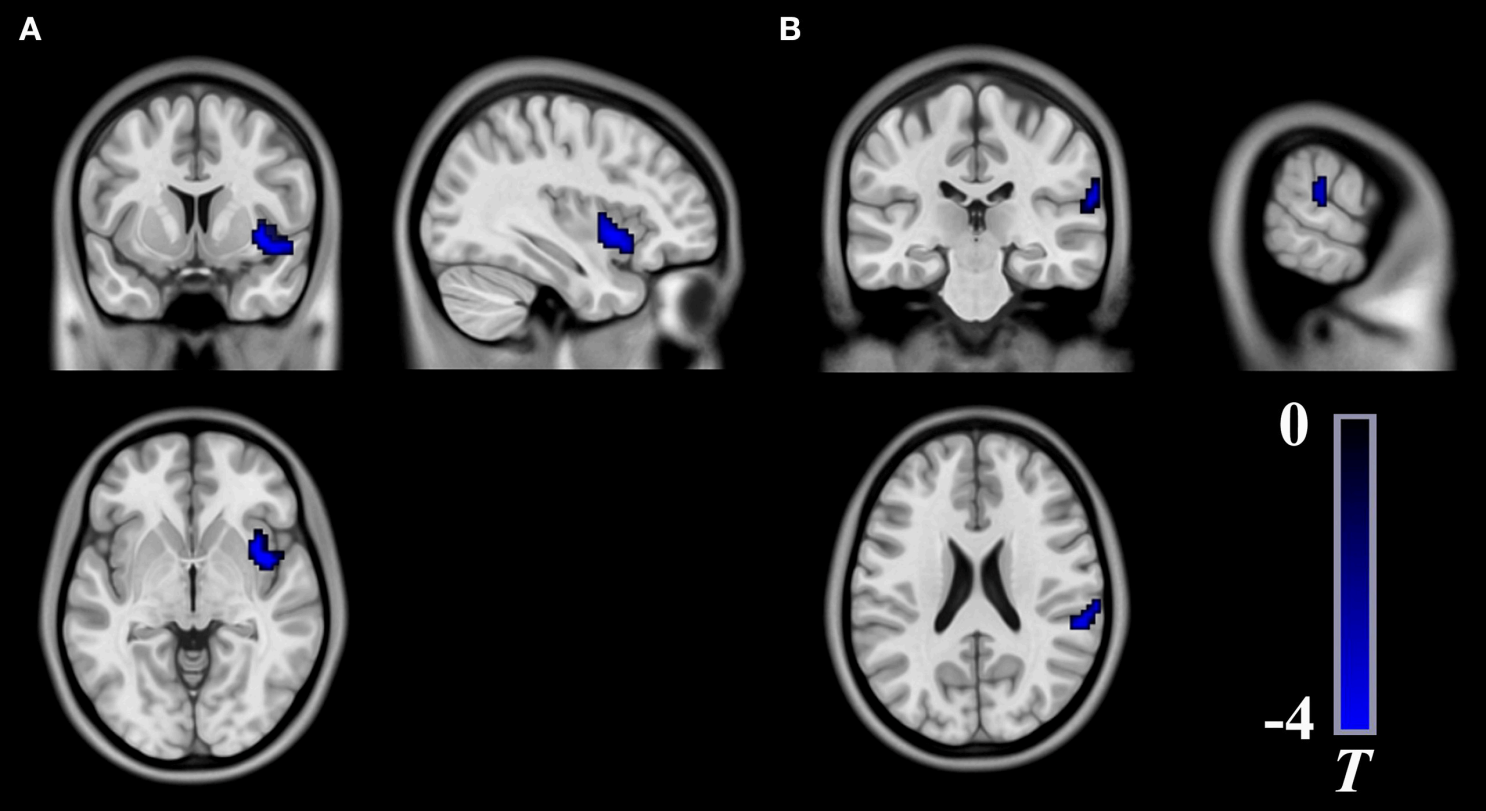
Brain regions show significant differences in long-range rsFCS **(A)** and short-range rsFCS **(B)** between T2DM patients and healthy controls. The color regions represent weaker rsFCS in patients with T2DM. The color bar indicates the *T*-value from two-sample *t*-tests. rsFCS, resting state functional connectivity strength.

**Figure 2:**
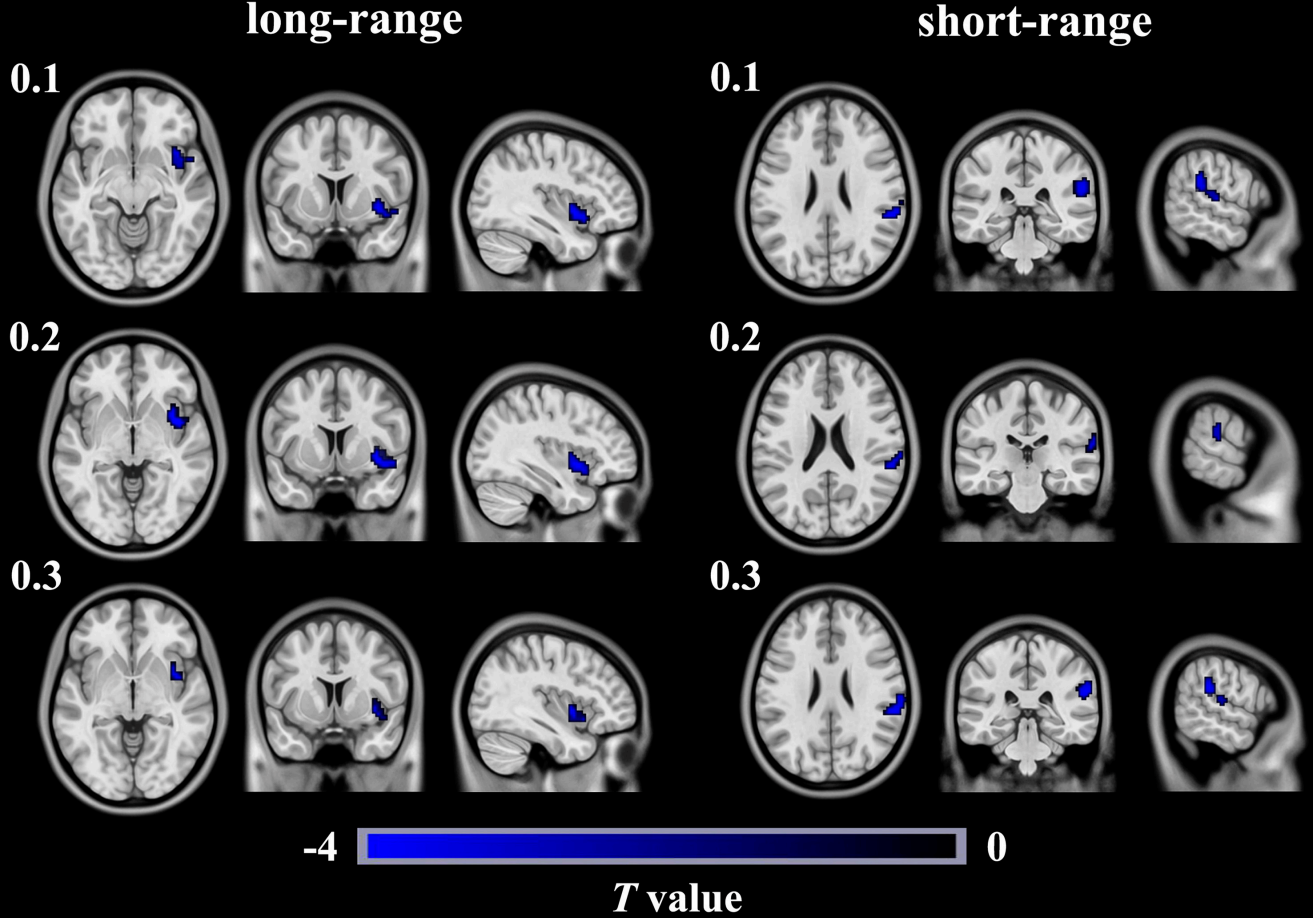
Brain regions show significant differences in long- and short- rangersFCS between T2DM patients and healthy controls in different thresholds of 0.1, 0.2, and 0.3. The color regions represent weaker long- or short- range rsFCS inpatients with T2DM than in healthy controls. The color bar indicates the *T*-value from two-sample *t*-tests. rsFCS, resting statefunctional connectivity strength.

Compared with the HCs, the T2DM patients exhibited weaker long-range rsFCS in the right insula(AlphaSim correction, cluster >40, cluster level *P* < 0.05), and weaker short-range rsFCS in the right supramarginalgyrus (SG) (AlphaSim correction, cluster >48, cluster level *P* < 0.05) after controlling for the age, gender and years of education (Table 2, Figure 1). These results were further verified to be reliable with the rsFCS analysis at the thresholds of 0.1 and 0.3 (Figure 2). The brain regions with significant intergroup difference in global-range rsFCS are highly overlapped with those in long-/short-range rsFCS (please see Supplementary Material).

**Table 2:** Brain regions with weaker long-and short-range FCS in T2DM.

**Regions**	**Peak (MNI)**			**Number of voxels**	*T*-value^*^
	**x**	**y**	**z**		
**LONG-RANGE FCS**					
Right insula	39	3	−3	81	−4.475
**SHORT-RANGE FCS**					
Right supramarginal gyrus	60	−33	24	67	−3.873

*FCS, functional connectivity strength; MNI, Montreal Neurological Institute; X, Y, Z, coordinates of primary peak locations in the MNI space*;

* *T-value of two sample t-test (P < 0.05, AlphaSim correction)*.

### Intergroup differences in seed-based FC

**Figure 3:**
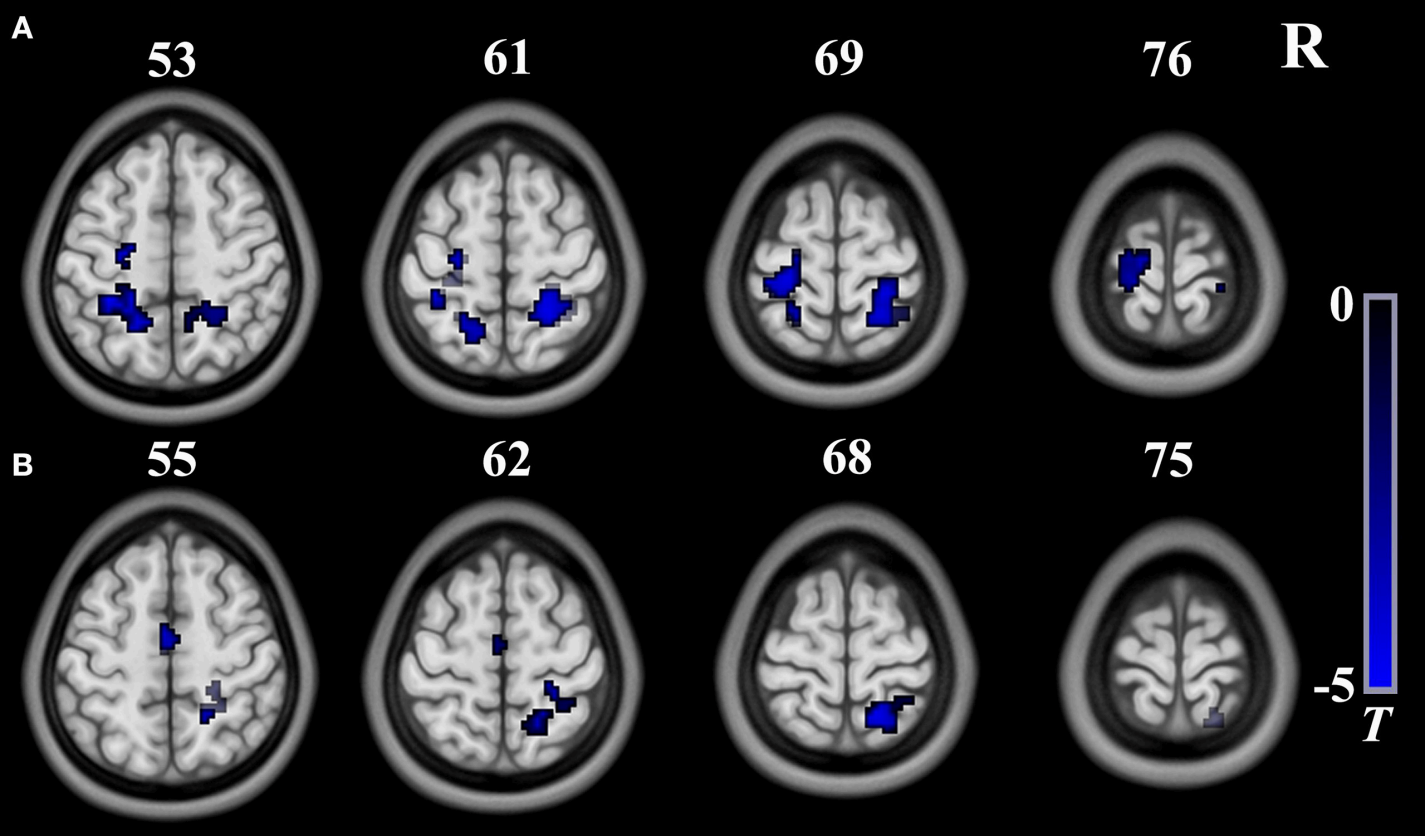
Brain regions show significant intergroup differences in seed-based FC. **(A)** The seed is the right insula from long-range rsFCS comparison; **(B)** The seed is theright supermarginal gyrus from short-range rsFCS comparison. The color regions represent decreased FC in patients. The color bar indicates the *T*-value from two-sample *t*-tests. FC, functional connectivity; rsFCS, resting state functional connectivity strength.

As shown in Table 3 and Figure 3, the FC between the right insula and the left postcentral/precentral gyrus, the left superior parietal lobule (SPL) and right postcentral gyrus/SPL (long-range FC) decreased in T2DM patients compared with the HCs (AlphaSim correction, cluster >18, cluster level *P* < 0.05). Additionally, the T2DM patients have decreased FC between the right SG and the bilateral supplementary motor area (SMA), the right postcentralgyrus and the right SPL (short-range FC) (AlphaSim correction, cluster >21, cluster level *P* < 0.05).

**Table 3:** Brain regions with decreased seed-based functional connectivity in T2DM.

**Seed ROIs**	**Target ROIs**	**Peak (MNI)**			**Number of voxels**	*T* value^*^
		**x**	**y**	**z**		
Right insula	Left postcentral/ precentral gyrus and	−33	−39	54	207	−4.859
	Superior parietal lobule					
	Right postcentral gyrus/superior parietal lobule	24	−48	57	167	−5.159
Right supramarginal gyrus	Bilateral supplementary motor area	9	−3	57	43	−4.065
	Right postcentral gyrus	33	−45	63	24	−3.612
	Right superior parietal lobule	15	−54	66	31	−4.337

*MNI, Montreal Neurological Institute; X, Y, Z, coordinates of primary peak locations in the MNI space*;

* *T-value of two sample t-test (P < 0.05, AlphaSim correction)*.

### Correlations between the rsFCS/seed-based FC and clinical/cognitive variables

**Figure 4:**
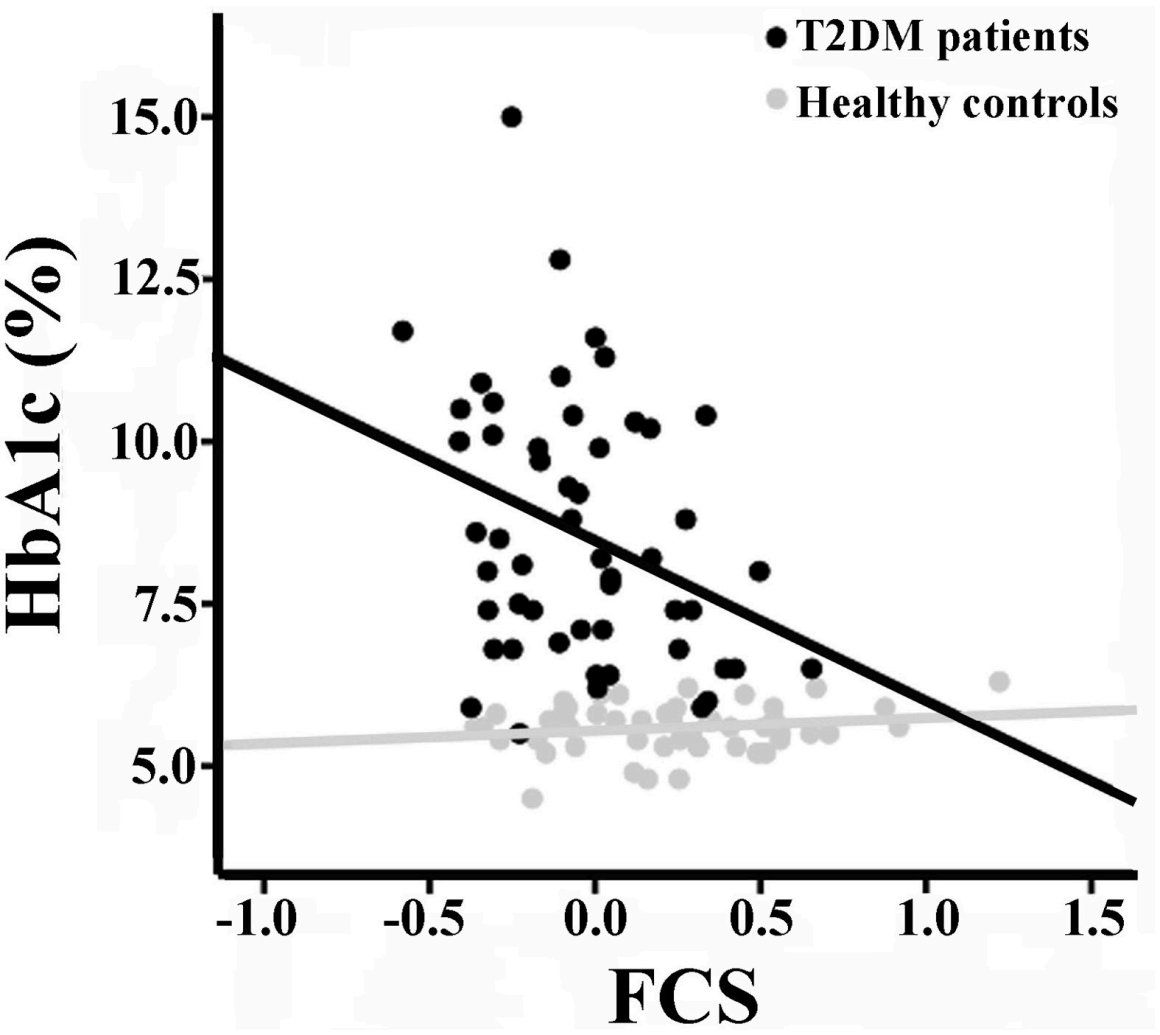
Correlation between the long-range rsFCS of right insula and HbA1c inboth groups. There is significant negative correlation in T2DM patients (*r* = −0.361, *p* = 0.046), but no correlation in healthy controls (*r* = 0.155, *p* = 0.327). There was no significant difference in correlation coefficients between the two groups (*z* = 1.12, *P* = 0.263). rsFCS, resting state functional connectivity strength.

**Figure 5:**
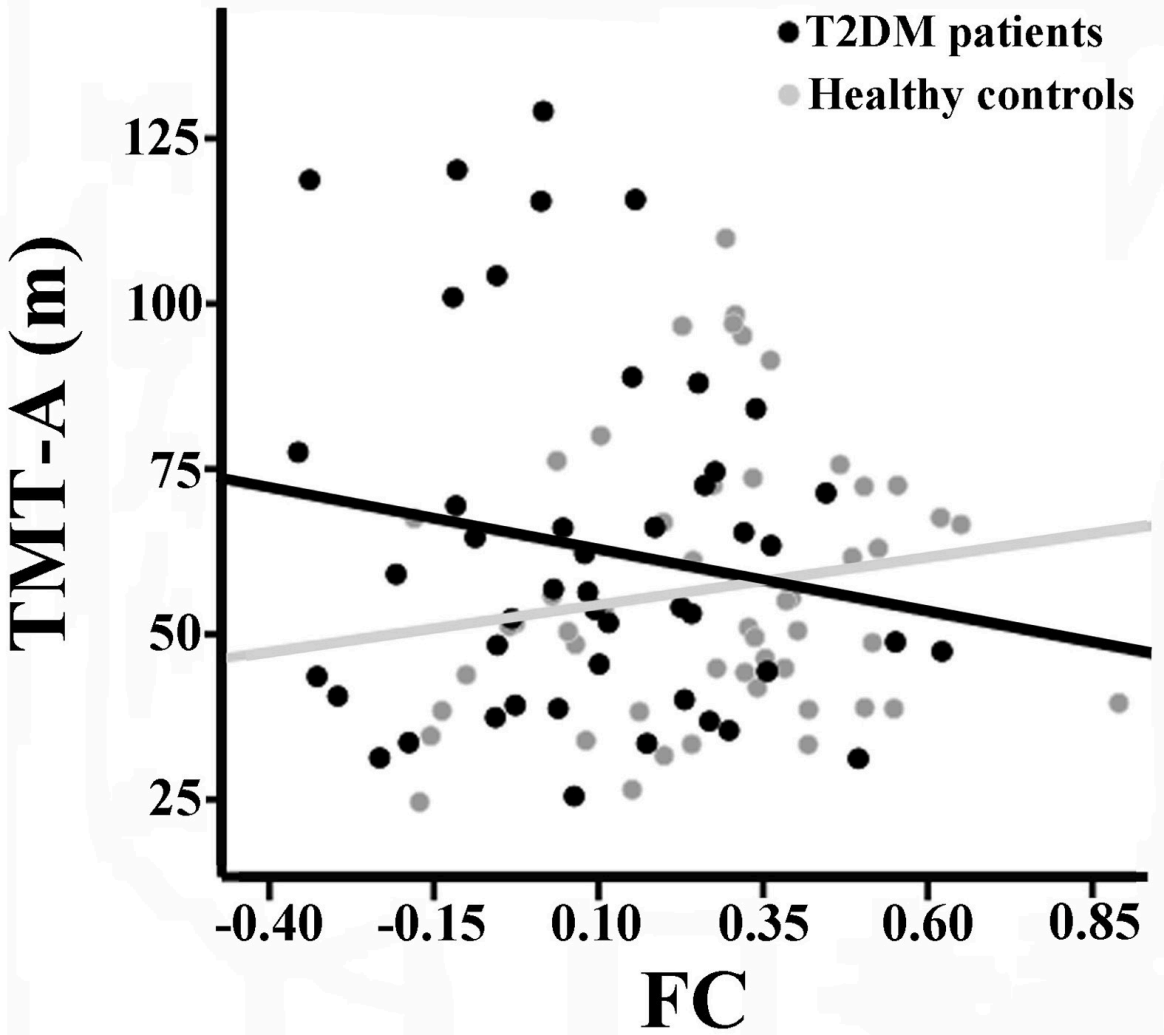
Correlation between TMT-A scores and the FC of the right SG with bilateral SMA in the two groups. There is significant negative correlation in T2DM patients (*r* = −0.436, *p* = 0.014), but no correlation in healthy controls (*r* = 0.071, *p* = 0.632). There was significant intergroup difference in the correlation coefficients (*z* = −2, *P* = 0.046). FC, functional connectivity strength; SG, supramarginal gyrus; SMA, supplementary motor area.

There were no significant correlations between the rsFCS/seed-based FC and clinical/cognitive variables in the HCs (*P* > 0.05). Significant negative correlation was found between the long-range rsFCS of the right insula and HbA1c levels in T2DM patients (*r* = −0.361, *P* = 0.046; Figure 4). There was no significant difference in correlation coefficients between the two groups (*z* = 1.12, *P* = 0.263). Besides, there were no correlations between rsFCS and cognitive variables. The FC between the right SG and the bilateral SMA negatively correlated with TMT-A scores in T2DM patients (*r* = −0.436, *P* = 0.014; Figure 5). Moreover, the coefficient of the T2DM patients was lower than that of the healthy controls (*z* = −2, *P* = 0.046). However, when Bonferroni correction was performed for multiple comparisons, no correlation survived.

### Intergroup differences in GM volumes

Voxel-wise comparisons revealed no significant difference in GMV within the whole brain between the two groups (AlphaSim correction, cluster >230, cluster level *P* < 0.05).

## Discussion

So far as we know, this is the first study to investigate both long-range and short-range rsFCS in patients with T2DM. The T2DM patients exhibited weaker long-range rsFCS in the right insula and weaker short-range rsFCS in the right SG. Seed-based FC analysis revealed weaker FC between the right insula and the left postcentral/precentral gyrus, the left SPL and right postcentral gyrus/SPL, and between the right SG and the bilateral SMA, the right postcentral gyrus and the right SPL in the T2DM patients. In the T2DM patients, the long-range rsFCS in the right insula was negatively correlated with HbA1c levels; and the FC between the right SG and the bilateral SMA was negatively correlated with TMT-A scores.

### Long-range rsFCSalteration in T2DM patients

In our results, the T2DM patients exhibited weaker long-range rsFCS in the right insula, and weaker FC between the right insula and the left postcentral/precentral gyrus, the left SPL and the right postcentral gyrus/SPL. The insula plays a crucial role in many cognitive functions, such as time processing (Kosillo and Smith, 2010), recognition memory (Bermudez-Rattoni, 2014), decision making (Smith et al., 2014) and speech production (Ackermann and Riecker, 2010). Previous studies have shown that the anterior insula is a central area for controlling human high-level cognition, such as attention processing (Brass and Haggard, 2010; Menon and Uddin, 2010; Nelson et al., 2010). The postcentral gyrus belongs to the somatosensory cortex, which participates in somatosensory stimulus processing, and involves attention processing by cooperating with the insula (Chen et al., 2010). Right anterior insula and precentral gyrus/SPL were also activated during performing attention task (Touroutoglou et al., 2012). Additionally, anterior insula cortex and somatosensory cortices are also involved in top-down and bottom-up information integration (Gu et al., 2013). Our study revealed the rsFCS in the right insula was negatively correlated with the HbA1c levels in T2DM patients, although cannot survived the multiple comparisons correction, which means higher level of blood glucose impacts the insular rsFCS. The correlation between the HbA1c levels and brain FC has also been reported in another study (Xia et al., 2015b). Although, previous studies have reported that high HbA1c levels correlated with cognition decline in T2DM patients (Allen et al., 2004; Zhou et al., 2010; Hui et al., 2015), ourT2DM patients exhibited no obvious cognitive impairments, which demonstrated that the rsFCS alteration in the right insula occurred before the obvious cognitive decline in T2DM patients. Therefore, it is plausible to speculate that the T2DM patients would exhibit impairments in insula-related cognitive functions with disease's development and occurrence of poor blood glucose control.

### Short-range rsFCSalteration in T2DM patients

As an important part of inferior parietal lobule, SG is considered to be a pivotal joint area of the temporal, parietal, and occipital lobes (Hagmann et al., 2008). SG is involved in various cognitive processes, particularly in memory, language and attention (Deschamps et al., 2014; Humphreys and Ralph, 2015). In our study, weaker short-range rsFCS was observed in the right SG, which was attributed to the reduced FC between the right SG and the bilateral SMA, the right postcentral gyrus/SPL. A previous research showed that the SG had positive connectivity with SMA, postcentral gyrus and SPL in healthy volunteers and these areas were involved in sensorimotor processing and visual attention (Zhang and Li, 2014). Another study showed that in healthy people, the activation of SPL was correlated with attention switching ability in a distractor condition (Bledowski et al., 2004). Additionally, SG and SMA were activated during visual attention tasks (Linden et al., 1999) and the SPL mediated the selection biases in visual search tasks in healthy subjects (Pollmann et al., 2003). Liang et al. (2012) had also revealed decreased connectivity between SG and SPL in patients with mild cognitive impairment, which was related to attention regulation impairment. These studies demonstrated that the SG and its connectivity with SMA and SPL have important role in attention processing. Previous studies have shown that cognitive decline in patients with T2DM is mainly manifested in information-processing speed, memory, executive function and attention (Gregg et al., 2000; Hassing et al., 2004; Kanaya et al., 2004). In our study, significant negative correlation was observed between the TMT-A scores and the FC of the SG with the bilateral SMA (uncorrected). The TMT is a test of visual conceptual and visuo-motor tracking, and the TMT-A purportedly measures attention and visual search and motor function (Crowe, 1998). Our correlation results possibly indicated that altered FC of the SG with the bilateral SMA was associated with the performance of attention and visual search and motor function in T2DM patients.

Here we noticed that no correlation was found between rsFCS index and cognitive assessment, which may partially attribute to the different algorithm of rsFCS and FC. For the rsFCS algorithm, it is a summation of the connections between a given voxel and all other voxels. And for the FC, it evaluates the connection between the seed area and other voxels. FCS and FC reflect the different properties and different dimensions of functional connectivity. The correlations between the FC and imaging index may not exist between the FCS and imaging measures. Additionally, altered rsFCS may not have the direct associations with the imaging measures.

In this study, the T2DM patients exhibited weaker long- and short-range rsFCS. Previous researches have demonstrated that the long-range FC provides quick link between remote brain regions (Achard et al., 2006), and plays vital role in conducting cognitive function by traveling across multiple modules and realizing the information integration (He et al., 2008; Bullmore and Sporns, 2012). Different from the long-range FC, the short-range FC is lower metabolic and time costs so that predicted stronger functional connectivity between neighboring brain regions (Raymond et al., 2005). In a study by Dai et al. (2015) reported that long-range FC is easy to be damaged in patients with Alzheimer's disease. However, our results demonstrated T2DM patients had both long- and short-range rsFCS alterations and did not exhibit dominant long-range rsFCS change like that in Alzheimer's disease. Given long- and short-range FC have different physiological basis and functions, the difference between our and Dai's results might be attribute to the different pathophysiological basis of the two diseases.

There are still some notable limitations. First, this study is a cross-sectional study, which could not monitor the dynamic alterations in cognitive functions of the T2DM patients. A follow up study would be necessary to evaluate the value of rsFCS in predicting the cognitive decline in T2DM patients. Second, the neuropsychological tests used in this study were not comprehensive and sensitive enough and may cannot detect some subtle cognitive impairments. Third, the long- and short-range rsFCS cut off point of 75 mm is arbitrary to some extent. Finally, the T2DM patients in our study received treatment with different methods; treatment-related effect should be noted as a limitation.

## Conclusion

To the best of our knowledge, this is the first study to evaluate the long- and short-range rsFCS among whole gray matter in T2DM patients. The T2DM patients exhibited weaker long-range rsFCS in the right insula and weaker short-range rsFCS in the right SG. Our results indicate that the rsFCS alteration occurs before obvious cognitive deficits and may be helpful for understanding the neuromechanism of cognitive declines in T2DM patients.

## Author contributions

LL, YZ, and QZ designed research; LL, YZ, WQ, SL, and QZ performed research; LL and YZ were involved in the clinical assessment; LL, YZ, WQ, and QZ analyzed data; LL, YZ, WL, and QZ wrote the paper.

### Conflict of interest statement

The authors declare that the research was conducted in the absence of any commercial or financial relationships that could be construed as a potential conflict of interest.
