# Supplementary material for: Weaker Functional Connectivity Strength in Patients with Type 2 Diabetes Mellitus
Source: Front Neurosci. 2017 Jul 7;11:390. doi: 10.3389/fnins.2017.00390 (PMC5500656; doi:10.3389/fnins.2017.00390)
Supplement: Supplementary file 1 [file DataSheet1.DOCX]

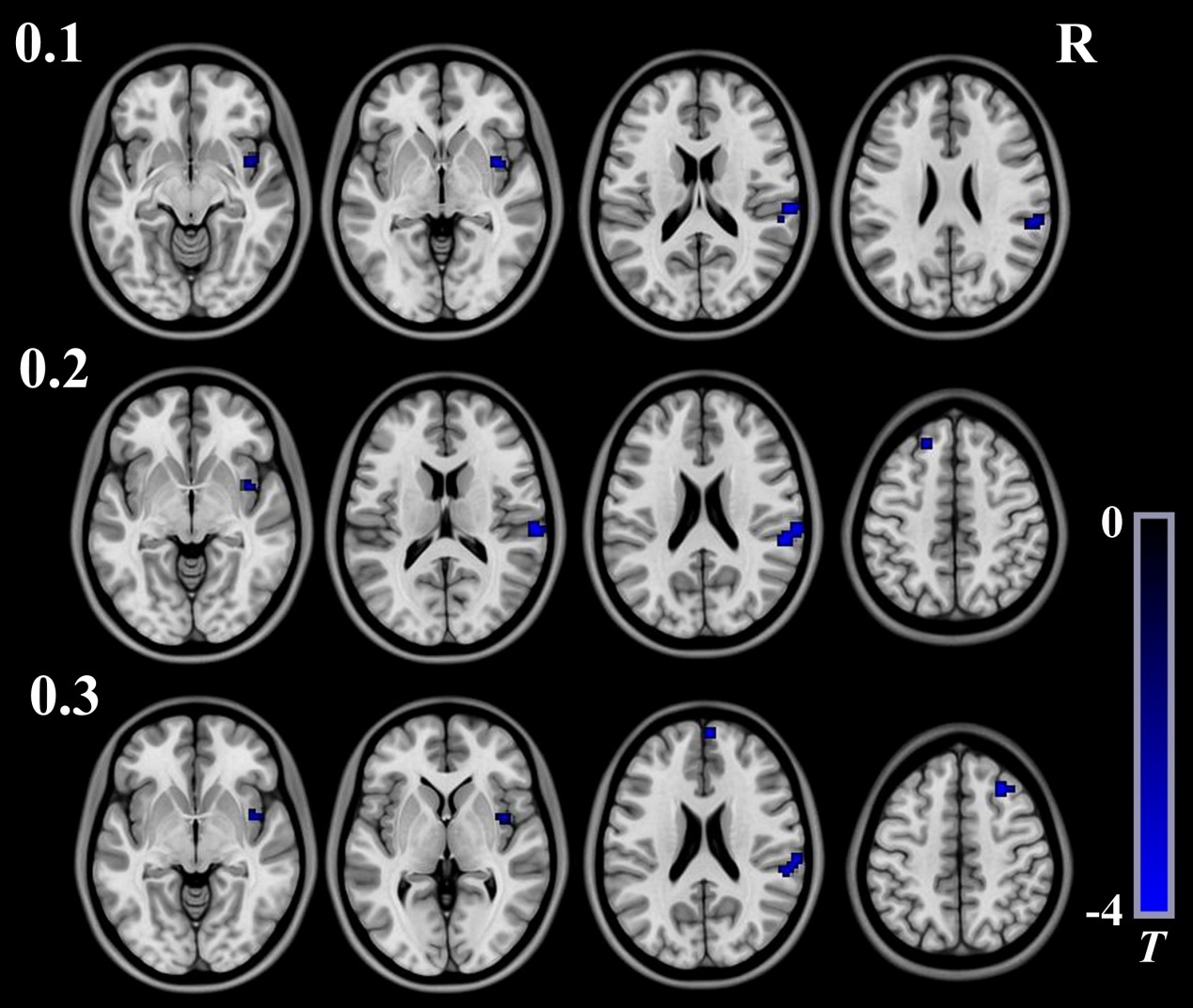


**Figure S1. Brain regions show significant differences in global-range rsFCS between T2DM patients and healthy controls in different thresholds of 0.1,0.2 and 0.3.**The color regions represent weaker global-range rsFCS in patients with T2DM compared with healthy controls (AlphaSim correction, cluster >18 voxels, cluster level *P* < 0.05).
